# The draft genome assembly and annotation of allotetraploid *Festuca glaucescens*

**DOI:** 10.1186/s12870-025-07558-8

**Published:** 2025-11-18

**Authors:** Izabela Pawłowicz, Piotr Kopeć, Arkadiusz Kosmala

**Affiliations:** 1 Plant Physiology Team, Institute of Plant Genetics, Polish Academy of Sciences, Strzeszynska 34, Poznan, 60-479 Poland; 2 Department of Computational Biology, Faculty of Biology, Adam Mickiewicz University, Umultowska 89, Poznan, 61-614 Poland

**Keywords:** *Festuca glaucescens*, Genome assembly, Polyploidy, Repeatome, Gene annotation, Functional genomics, Forage grasses

## Abstract

**Background:**

We present the first long-read genome assembly and annotation for *Festuca glaucescens*, a member of a genus characterized by polyploidy, large genome sizes, and a high content of repetitive sequences. Fescues are important forage and turf grasses in temperate regions, valued for their resilience to abiotic stress. Though, the allotetraploid *F. glaucescens* is particularly notable for its drought tolerance, yet its genomic architecture has remained largely unexplored.

**Results:**

The haploid assembly spans 5.52 Gb, with > 98% gene space completeness as assessed by BUSCO. Repetitive elements comprise ~ 77% of the genome, dominated by Gypsy and Copia LTR retrotransposons. We predicted 72,385 protein-coding genes, the majority of which are supported by transcriptomic and homology-based evidence. While some contig-level fragmentation persists, the assembly successfully captures the gene-rich, repeat-dense landscape of this complex polyploid genome.

**Conclusions:**

This is the first annotated reference genome for a *Festuca* species, offering a foundational resource for functional genomics, comparative studies, and forage grass improvement.

**Supplementary Information:**

The online version contains supplementary material available at 10.1186/s12870-025-07558-8.

## Background

*Festuca* L. genus, comprising over 600 species, is one of the largest within the Poaceae family [1–4]. It belongs to the subtribe Loliinae, which is subdivided into the ‘broad-leaved’ (BL) and ‘fine-leaved’ (FL) evolutionary lineages. Members of Loliinae are distributed worldwide, particularly in temperate zones and tropical mountain regions [5–9].

Within the BL clade, a paraphyletic subgenus *Schedonorus* can be distinguished, exhibiting strong phylogenetic affinity with members of the genus *Lolium* [10–12]. Due to its monophyletic nature [5, 6, 13–15], and the ability to form spontaneous intergeneric hybrids, this group is often referred as the *Schedonorus–Lolium* complex or the broad-leaved *Schedonorus–Lolium* lineage.

Although taxa within the *Schedonorus–Lolium* complex share a uniform base chromosome number (x = 7), the two genera differ markedly in ploidy levels. It is estimated that approximately 70% of *Festuca* species are polyploid [16–18], a trend also observed in subgenus *Schedonorus*, which includes diploid, tetraploid, hexaploid, and octoploid taxa. In contrast, *Lolium* is a small genus comprising ten diploid species [1, 19].

Most *Schedonorus* representatives are obligate outcrossers, whereas both inbreeding and outcrossing mating systems are found in *Lolium*. *Festuca* species typically inhabit meadows and alpine grasslands [8], but also thrive in more extreme environments such as wetlands, mountains, and xeric ecosystems, reflecting their high tolerance to abiotic stresses. By contrast, *Lolium* species are known for their high yield, rapid growth, and forage quality, but are generally more susceptible to abiotic stresses. Both *Festuca* and *Lolium* include species of major agricultural importance, such as *F. pratensis*,* F. arundinacea*,* L. perenne* and *L. multiflorum* [5, 8, 20, 21]. 

*Festuca glaucescens* (*F. arundinacea* Schreb. subsp. Fenas (Lag.) Arcang.) (2n = 4x = 28) is an allotetraploid species containing two subgenomes, designated as G1 and G2 (G1G1G2G2) [14, 22–24]. It belongs to the so-called European clade of the subgenus *Schedonorus* [5, 25, 26], and is primarily found in the Mediterranean region. It is considered one of the phylogenetically oldest species within Loliinae and a likely progenitor (donor of the G subgenomes) to polyploid taxa such as the Continental and Rhizomatous morphotypes of hexaploid *F. arundinacea* [14, 23, 27, 28], as well as octoploid *F. atlantigena* [27]. It is closely related to *F. mairei*, and there is strong evidence that both species share a common ancestor – the diploid *F. scariosa* [29, 30].

*Festuca glaucescens* exhibits high tolerance to both drought and heat stress [31, 32]. During summer periods of water deficit, it enters a state of ‘quiescence’, characterized by a slowdown in metabolism and temporary cessation of growth, both of which are reversible upon rehydration [31]. Owing to these adaptive traits, *F. glaucescens* has been employed in backcross breeding programmes aimed at improving drought and heat tolerance in *L. multiflorum* [32]. A comprehensive physiological and molecular analysis of drought response further demonstrated that the species’ antioxidant capacity plays a central role in its stress resilience, with catalase activity identified as a key contributor [33].

Despite its physiological significance, *F. glaucescens* remains one of the least studied species within the genus *Festuca*. Here, we present the first long-read draft genome assembly, along with its annotation and repeatome analysis. This is also the first available genomic sequence for any *Festuca* species.

## Results and discussion

### Genome size estimation and ploidy assessment

**Fig. 1:**
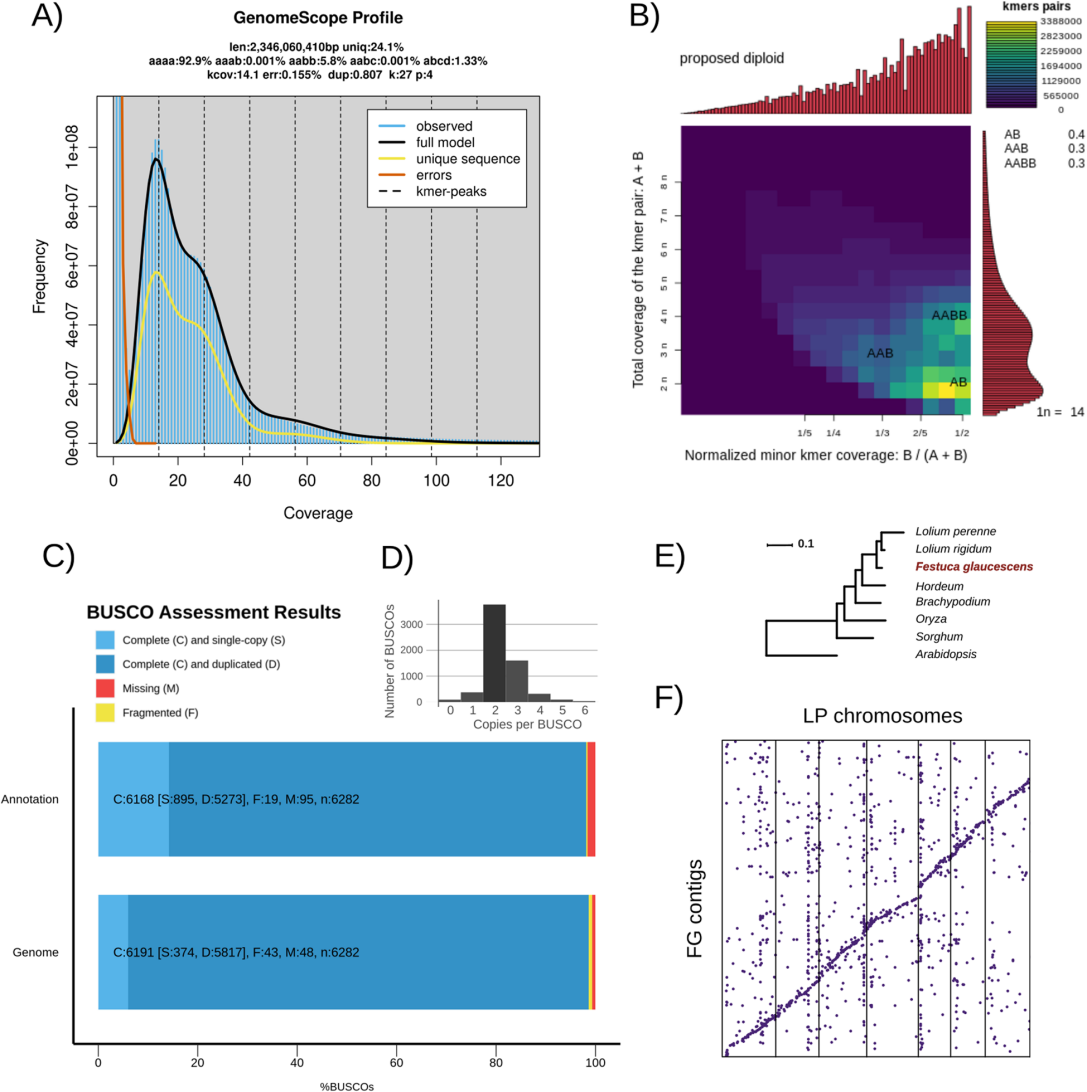
Genome size, ploidy structure, assembly completeness, and phylogenetic placement of *F. glaucescens*. **a** 27-mer frequency distribution from Illumina WGS data (GenomeScope2) with estimated monoploid genome size (~2.34 Gb). **b** Smudgeplot confirming allotetraploidy, with AB, AAB, and AABB configurations and inferred haploid chromosome number (n = 14). **c ** BUSCO completeness of contig assembly (98.6%) and gene set (95.6%) against *poales_odb12*. **d **BUSCO copy-number distribution, with most genes occurring twice, consistent with an allotetraploid genome, and a notable fraction occurring in three or more copies, reflecting residual uncollapsed haplotigs. **e **Species tree inferred by OrthoFinder from 7,953 orthologs. Scale bar indicates substitutions per site. **f **Whole-genome alignment of *F. glaucescens* contigs to the *L. perenne* reference genome showing broad coverage across all chromosomes

To estimate the genome size of *F. glaucescens*, k-mer profiling was performed using Illumina whole-genome sequencing (WGS) data from genotype Fg1. Prior to analysis, adapter sequences were trimmed, and reads mapping to organellar genomes (*F. arundinacea* chloroplast and *L. perenne* mitochondrion) were filtered out, representing approximately 2% of the dataset. The remaining reads were used to generate a 27-mer histogram, which was analyzed using GenomeScope2 under a tetraploid model (Fig. 1a).

The monoploid genome size (1Cx) of Fg1 was estimated at 2.34 Gb. This estimate is consistent with previously reported cytometric measurements: 2.6 Gb (2.7 pg) for *F. glaucescens* and 2.4 Gb (2.43 pg) for the closely related tetraploid *F. mairei* [34]. It also corresponds reasonably well with the value available in the Plant DNA C-values database, which reports a 1 C genome size of 4.2 Gb for *F. glaucescens*. In tetraploid species, the 1 C value represents the DNA content for an unreduced gamete (2x), and is therefore expected to be roughly double the monoploid genome size estimated after sequencing. These values are in reasonable agreement, with the ~ 9% discrepancy likely reflecting methodological differences between sequencing-based estimates and cytometry.

Comparable genome sizes have also been reported for closely related *Lolium* species including *L. perenne* [34–39] and *L. ridigum* [40]. Interestingly, the monoploid genome size of both diploid and tetraploid *F. pratensis* is around 3.2 Gb, whereas that of hexaploid *F. arundinacea* is slightly smaller, ~ 2.8 Gb [19, 34, 35]. These observations align with the broader pattern observed in the Loliinae subtribe, where genome sizes range widely, from 2.6 Gb/1 C to 11.8 Gb/1C [19, 35, 41], and are closely tied to variation in ploidy levels. Comparison of genome sizes between *F. glaucescens*,* F. pratensis* and *F. arundinacea* provide partial support for the hypothesis of genome size reduction in higher polyploids within Loliinae [41, 42].

The k-mer frequency profile showed a complex distribution in polyploid genomes. In particular, the strong prevalence of the *aabb* configuration over *aaab* in the GenomeScope output suggests a disomic inheritance pattern, consistent with the behavior of allotetraploid genomes. Smudgeplot analysis further supported this interpretation, showing distinct peaks corresponding to AB, AAB, and AABB k-mer combinations at relative frequencies of 0.4, 0.3, and 0.3, respectively (Fig. 1b). These results are consistent with a genome composed of two diverged subgenomes and confirm the allotetraploid status of *F. glaucescens*. The inferred haploid chromosome number (*n* = 14) aligns with cytogenetic observations in the *Festuca–Lolium* complex, which exhibits a base chromosome number of x = 7 (Fig. 1b). The allopolyploid nature of *F. glaucescens* was first suggested based on cytological evidence [43–45]. This has since been corroborated by multiple lines of evidence, including genomic in situ hybridization (GISH) [23], restriction fragment length polymorphism (RFLP) analysis [22], and ribosomal DNA (rDNA) analysis [30, 46].

### *De novo* genome assembly

To conduct *de novo* genome assembly for *F. glaucescens*, we generated approximately 59.6 Gb of PacBio Hi-Fi data, corresponding to ~ 6.5× haploid coverage based on a monoploid genome size of 2.3 Gb per subgenome. Assembly was performed with hifiasm using internal purging, aiming to collapse haplotypes into a primary representation while retaining divergent homeologous sequences in order to reflect the allotetraploid structure of the genome. The resulting assembly spans 5.52 Gb across 13,136 contigs, with an N50 of 872,590 bp, a mean contig length of 420 kb, and a maximum contig length of 29.4 Mb (Table 1). The GC content is 45.44%. Notably, the assembly contains no gaps, indicating complete base-level coverage across contigs.

While contig-level fragmentation remains, these metrics fall within the expected range for a draft assembly of a large, repeat-rich, and polyploid grass genome. The total assembly size slightly exceeds the estimated haploid genome size, suggesting the presence of uncollapsed haplotigs (see Supplementary Note).

**Table 1:** Summary statistics for *F. glaucescens* genome assembly

Feature	Value
Total length (bp)	5,536,934,307
Number of contigs	13,482
Mean contig size (bp)	410,690
The largest contig size (bp)	29,439,325
N50 (bp)	870,179
L50 (bp)	1,646
GC content	45.44%

Assembly completeness was assessed using BUSCO with the *poales_odb12* dataset. A total of 98.6% of BUSCO genes were identified as complete, with 6% present in single copy and 92.6% duplicated (Fig. 1c). Only 0.7% were fragmented, and 0.8% were missing. These values indicate high gene space completeness, with duplication rates consistent with expectations for an allotetraploid genome. In addition, whole-genome alignment against the *L. perenne* reference genome revealed that *F. glaucescens* contigs collectively align along the full length of all *L. perenne* chromosomes (Fig. 1f), suggesting broad syntenic coverage despite the fragmented nature of the assembly.

To further assess the structure of the *F. glaucescens* assembly, we mapped PacBio HiFi reads back to the contigs and analyzed read depth in conjunction with k-mer multiplicity profiles (Supplementary Fig. 1). A clear bimodal coverage distribution was observed, with one subset of contigs showing modal coverage in the 6–7× range—consistent with the expected depth per haplotype—and lower k-mer multiplicity, suggesting the presence of uncollapsed haplotigs. In contrast, longer contigs showed higher coverage and accumulated more sequence depth, contributing disproportionately to the total assembly size. These patterns suggest that, despite internal purging by HiFiAsm, the final assembly (5.52 Gb) still retains some redundant haplotig-like sequences. This interpretation is further supported by the BUSCO copy-number distribution (Fig. 1 d), which shows a notable fraction of genes present in three or more copies, consistent with residual haplotigs. Overall, the excess assembly size relative to genome size estimates (~ 4.6 Gb) is best explained by redundancy from uncollapsed haplotigs, reflecting the limits of the modest long-read coverage (~ 6.5× per haplotype), which was insufficient for complete haplotype resolution.

To evaluate whether further purging was feasible, we performed an exploratory run of *purge_dups* on the unpurged *Hifiasm* assembly (Supplementary Fig. 2). While the tool identified contigs with lower depth and multiplicity as potential duplicates, the resulting purged assembly was reduced to 3.72 Gb – substantially below the estimated genome size, indicating likely overpurging. BUSCO analysis further confirmed this, showing that while both assemblies are dominated by two-copy BUSCOs, the purged version exhibited a strong increase in single-copy BUSCOs (1611 vs. 374) and higher missing rates (2.3% vs. 0.8%), consistent with the collapse of true homeologous copies (Supplementary Fig. 3). Furthermore, the classification of contigs as duplicates showed extensive overlap with those retained as primary (Supplementary Fig. 4), suggesting that *purge_dups* could not reliably distinguish true haplotigs from homeologous sequences in this context. Based on this evidence, we retained the original *Hifiasm* primary assembly, which applies conservative internal purging and better preserves gene content and subgenomic structure.

By contrast, chromosome-scale genome assemblies with much deeper coverage have been reported for related species, including *L. perenne* (Lolium_2.6.1) [37, 47], and *L. rigidum* [40]. Notably, a haplotype-resolved assembly capturing both haplotypes has been achieved for *L. multiflorum* [48]. While our coverage was sufficient for constructing a high-quality draft assembly, it was not adequate for chromosome-scale phasing or aggressive haplotig filtering. As such, the final assembly may retain some redundancy from uncollapsed haplotigs, but this approach prioritizes completeness and preservation of subgenomic structure, providing a more accurate representation of the allotetraploid genome.

### Gene annotation and structural features

Gene annotation was performed using the EASEL pipeline, which integrates transcriptomic, protein homology, and ab initio evidence to produce high-confidence gene models. In total, 12.62 Gb of RNA-seq data were generated across multiple tissues and incorporated into the pipeline to support transcript structure prediction. This process resulted in the identification of 72,385 protein-coding genes and 110,990 transcripts (Table 2). Among these, 124 genes were located on contigs assigned to organellar genomes.

Assessment of annotation completeness using BUSCO indicated 95.6% completeness, with 13.2% of genes found in single copy and 82.4% duplicated, consistent with expectations for an allotetraploid genome.

Functional annotation showed that 66,350 genes had detectable homology to known proteins, while 6,035 genes had no significant match in public databases. Gene Ontology (GO) terms were assigned to 26,902 genes, and 65,706 genes contained at least one Pfam domain.

**Table 2:** Summary of gene annotation statistics for the *F. glaucescens* genome

Feature	Value
Number of genes	72,385
Number of transcripts	110,990
Number of exons	645,910
Number of single-exon genes	18,157
Mean transcripts per gene	1.5
Mean exons per transcript	5.82
Mean gene length (bp)	3,609
Median gene length (bp)	2,343
Mean CDS length (bp)	1,420
Mean exon length (bp)	244
Median exon length (bp)	133
Mean intron length (bp)	535
Median intron length	139

The number of predicted protein-coding genes in *F. glaucescens* exceeds those reported for diploid relatives such as *L. perenne* and *L. rigidum*, which typically range between 38,000 and 57,000 genes depending on assembly and annotation strategy. This increase is consistent with the expected retention of homeologous gene copies in allotetraploid genomes. However, direct comparisons should be made cautiously, as differences in annotation pipelines, transcriptomic support, and filtering criteria can significantly affect gene counts.

The final gene set had a mean length of 3,609 bp (median: 2,343 bp), with coding sequences averaging 1,420 bp. On average, genes consisted of six exons and five introns, and 18,157 genes were mono-exonic. The cumulative coding sequence length for the longest isoforms totaled 97.7 Mb, while intronic regions accounted for approximately 158 Mb. These structural features are broadly consistent with those observed in other large, repeat-rich grass genomes.

As a validation step, we ran OrthoFinder on the predicted *F. glaucescens* proteome alongside selected Poaceae species. The resulting species tree, inferred from gene orthogroup relationships, correctly grouped *F. glaucescens* with members of the *Lolium* clade, consistent with its known phylogenetic position (Fig. 1e). This supports the overall reliability of the annotation and orthology assignments.

### Repeat landscape

Repeat annotation revealed that interspersed repeats account for 77.1% of the *F. glaucescens* genome, closely matching the 76.3% estimated via k-mer analysis. Retroelements dominate the repeat landscape, comprising 50.8% of the genome. Long terminal repeat (LTR) retrotransposons are particularly abundant (47.1%), with *Gypsy/DIRS1* (27.5%) and *Ty1/Copia* (10.6%) representing the most prominent superfamilies (Table 3). Additional retroelements include long interspersed nuclear elements (LINEs, 3.5%), while short interspersed nuclear elements (SINEs) are relatively rare (0.1%).

DNA transposons constitute 4.8% of the genome, with *MULE-MuDR*,* hobo-Activator*,* and Tourist/Harbinger* being the most represented subclasses. Notably, 21.5% of the genome was classified as uncharacterized repetitive content, likely corresponding to lineage-specific or highly diverged repeat families absent from current databases.

**Table 3:** Summary of repetitive element content in the *F. glaucescens* genome based on repeatmasker annotations

	Number of elements	Length (bp)	Percentage of sequence
**Retroelements**	3,027,048	2,810,987,678	50.77
SINEs:	8,249	5,426,907	0.1
LINEs:	198,778	195,525,433	3.53
L2/CR1/Rex	9	1,850	0
RTE/Bov-B	17,370	12,043,956	0.22
L1/CIN4	170,426	178,802,655	3.23
LTR elements:	2,820,021	2,610,035,338	47.14
BEL/Pao	12,632	4,390,436	0.08
Ty1/Copia	664,120	584,359,667	10.55
Gypsy/DIRS1	986,248	1,522,519,945	27.5
Retroviral	36,188	15,142,101	0.27
**DNA transposons**	335,381	265,605,888	4.8
hobo-Activator	30,685	18,474,474	0.33
Tc1-IS630-Pogo	17,003	3,926,852	0.07
MULE-MuDR	60,303	43,183,975	0.78
PiggyBac	2,296	1,056,403	0.02
Tourist/Harbinger	50,414	27,574,586	0.5
Rolling-circles	12,057	13,497,810	0.24
**Unclassified**	3,033,042	1,192,324,644	21.53
**Total interspersed repeats:**		4,268,918,210 bp	77.10%
Small RNA:	14,810	17,516,461	0.32
Simple repeats:	284,515	13,154,813	0.24
Low complexity:	39,589	2,050,425	0.04

The overall repeat content of *F. glaucescens* is similar to that reported for *L. rigidum* [40] and significantly higher than in *L. perenne* [36, 37]. In all of these species, *Gypsy* and *Copia* LTR retrotransposons dominate the repeatome. This trend has also been observed in other comparative studies of *Festuca* and *Lolium* genomes across different ploidy levels, including *F. glaucescens* [34, 42].

According to Zwyrtková et al. (2020) [34], *Gypsy* elements are more abundant than *Copia* in *F. glaucescens*, *F. pratensis*, *F. mairei*, and *F. gigantea*. Moreover, high levels of unclassified LTR elements and LINEs were reported across these species, consistent with our findings. Interestingly, in *Lolium* species, the *Ty3/Gypsy Athila* lineage has been identified as the most abundant (up to 25% of the nuclear genome), suggesting a role in *Lolium* speciation and genome size expansion.

The study by Moreno-Aguilar et al. (2022) [42] also confirmed that repetitive elements occupy a substantial fraction of all studied *Festuca–Lolium* genomes, averaging over 50% of total genome size. Interestingly, a higher repeat content was observed in diploids (average: 68.7%) compared to higher polyploids (30.7%). This supports previous observations that polyploidization is often accompanied by repeat loss or differential proliferation, with LTR retrotransposons (especially *Angela* and *Athila*) being major contributors to genome size variation [49–51].

While previous studies have reported a reduction in repeat content with increasing ploidy, our results show that *F. glaucescens* retains a high proportion of repetitive elements despite its allotetraploid nature. This indicates that the relationship between ploidy level and repeat content is not straightforward and likely reflects lineage-specific evolutionary trajectories rather than a uniform pattern of repeat loss following polyploidization.

## Conclusions

We present the first comprehensive genome analysis of *F. glaucescens*, marking the first sequenced species within the genus *Festuca*. Our results provide a precise genome size estimate and confirm its allotetraploid nature. The draft assembly and accompanying gene annotation form a valuable foundation for downstream research, including transcriptomic analyses, candidate gene discovery, and trait mapping.

Although the assembly remains fragmented and does not resolve haplotypes, it captures a high proportion of conserved genes and encodes a structurally diverse, well-annotated gene set. This resource will support future work in *Festuca* and related grass species, particularly in the context of functional genomics and breeding. We believe that the presented details associated with *F. glaucescens* genome, being one of the ancestral progenitor of *F. arundinacea* could be especially valuable in understanding evolutionary history of these both species. *F. glaucescens* genome can underpin genome-wide association studies, QTL mapping, and marker-assisted selection in forage and turf grass breeding, particularly for traits linked to drought and heat tolerance. More broadly, its availability facilitates comparative and evolutionary genomics across polyploid *Festuca* species and will support further research aimed at improving agronomic performance and ecological adaptability in fescue and ryegrass cultivars.

Full subgenome resolution and haplotype phasing will require additional work, potentially involving deeper sequencing, long-range scaffolding (e.g., Hi-C), and parental or progeny data. Nevertheless, the current genome assembly provides a critical reference point for studying genome structure, gene function, and trait evolution in forage grasses. Future efforts building on this resource will enable chromosome-scale reconstruction and deepen our understanding of polyploid genome evolution in the *Festuca–Lolium* complex.

## Methods

### Plant material

The Fg1 genotype of *F. glaucescens* used in this study originated from the Institute of Plant Genetics Polish Academy of Sciences (Poznań, Poland). It has been established from a single seed, clonally propagated and maintained under greenhouse conditions. Seeds of *F. glaucescens* (accession ABY-Bn 354–1980) originated from the collection of the Institute of Biological, Environmental and Rural Sciences (IBERS, Aberystwyth, UK) [33]. Taxonomic identity and ploidy level of Fg1 were previously confirmed by fluorescence in situ hybridization (FISH), as described by Lechowicz et al. [33]. It revealed the presence of 28 chromosomes in *F. glaucescens* genome (metaphase plate) and a specific distribution of ribosomal DNA loci (5 S rDNA and 35 S rDNA), described earlier by Thomas et al. [46].

### DNA and RNA extraction and sequencing

Whole-genome and transcriptome sequencing were performed by Macrogen Europe (Amsterdam, Netherlands).

High-molecular-weight (HMW) genomic DNA was extracted from fresh leaf tissue using the Nanobind Plant Nuclei Big DNA Kit (Circulomics, Pacific Biosciences, USA), according to the manufacturer’s protocol. Whole-genome sequencing (WGS) was conducted using a combination of PacBio Sequel II and Illumina NovaSeq 6000 platforms. PacBio libraries (mean fragment length ~ 15 kb) were prepared using the SMRTbell Express Template Prep Kit 2.0 (PacBio), while Illumina paired-end libraries (~ 350 bp insert size) were generated using the TruSeq PCR-Free Kit (Illumina).

Total RNA was extracted from pooled tissue (leaves, stems, and roots) using the RNeasy Plant Mini Kit (Qiagen), followed by on-column DNase treatment with the RNase-Free DNase Set (Qiagen), following the manufacturer’s instructions. cDNA libraries were prepared using the TruSeq Stranded mRNA Library Prep Kit (Illumina) and sequenced on the Illumina NovaSeq X platform. We generated 83.5 million paired-end reads (12.6 Gb) from poly(A)-selected RNA libraries (2 × 151 bp). As part of the Easel v2.1.0-beta annotation pipeline, raw data were preprocessed with fastp, which retained 82.8 million reads (99.1%) after adapter and quality trimming. The resulting dataset comprised 12.5 Gb of high-quality bases, with a mean read length of 150 bp, 98.8% ≥Q20 and 96.1% ≥Q30, a GC content of ~ 52%, and a duplication rate of 16.3%. Processed reads were then aligned to the *F. glaucescens* genome using HISAT2, yielding an overall alignment rate of 84.8%.

### Genome size and ploidy estimation

To estimate the monoploid genome size and assess ploidy structure, k-mer profiling was performed using Illumina WGS reads. Prior to analysis, adapter sequences were trimmed, and reads mapping to known non-nuclear sequences were removed. Specifically, reads aligning to the *F. arundinacea* chloroplast genome (NCBI RefSeq accession: NC_028254.1) and the *L. perenne* mitochondrial genome (NC_036024.1) were filtered out using *bwa mem* v0.7.17-r1188 alignment [52]. These organellar reads represented approximately 2% of the dataset.

The remaining reads were used to generate a 27-mer frequency histogram using Jellyfish v2.2.10 [55]. The histogram was analyzed using *GenomeScope2* [53] under a tetraploid model to estimate haploid genome size, repeat content, and heterozygosity. Ploidy structure was further assessed using *Smudgeplot* v0.2.5 [53], based on the same k-mer profile.

### *De-novo* genome assembly

Genome assembly and initial quality control were carried out by Data2Biology (https://data2biology.com*)* and Xenstats (https://xenstats.com*)*, both based in Poznań, Poland.

PacBio HiFi reads were assembled de novo using *Hifiasm* v0.16.1-r375 [54] with default parameters, including internal purging of haplotigs. To evaluate the potential for further haplotig removal, we conducted a test run of *purge_dups* v1.2.5 [58]. on the unpurged *Hifiasm* assembly using default parameters. Gene space completeness was assessed with BUSCO v5.4.3, using the *poales_odb12* lineage dataset (*n* = 6,282) [55].

Assembled contigs were screened for organellar origin by aligning them to the *F. arundinacea* chloroplast genome (NCBI RefSeq: NC_028254.1) and the *L. perenne* mitochondrial genome (NC_036024.1) using BLASTn [56]. Contigs with ≥ 50% of their length aligning to either reference at an e-value threshold of 1e^−10^ were flagged as organellar.

Whole-genome alignments of *F. glaucescens* contigs to the *L. perenne* reference genome were performed with minimap2 v2.24 (options: *-x asm20*). Dot plots were generated using D-GENIES [57] and subsequently adjusted for clarity in Inkscape.

### Repeat annotation and genome masking

RepeatMasker v4.1.7-p1 with rmblast v2.14.1+ [58] was used to annotate and soft-mask the genome using the custom library (DOI: 10.5281/zenodo.15744278). The repeat library was generated with RepeatModeler v2.0.5 [63] using the Dfam 3.8 database [59]. Repeat prediction incorporated TRF v4.09 [60], RECON [61], and RepeatScout v1.0.6 [62]. Long terminal repeat (LTR) elements were identified using the LTRStruct module, which integrates GenomeTools v1.6.5 [63], LTR_Retriever [64], NINJA v1.0 (clustering only) [65], MAFFT v7.525) [66], and CD-HIT v4.8.1 [67], with the *ltrSeqLimit* parameter set to 11,000.

### Gene prediction and functional annotation

Gene prediction was performed on the soft-masked genome using the Easel v2.1.0-beta pipeline (https://gitlab.com/PlantGenomicsLab/easel), executed under Nextflow v24.10.3 [68]. Easel integrates multiple tools, including fastp [69] for read preprocessing, HISAT2 [70] for RNA-seq alignment, StringTie2 [71] for transcript assembly, AUGUSTUS [72] and GALBA [73] for ab initio prediction, and miniprot [74] for protein-genome alignments. Functional annotation was conducted using EnTAP [75], which leverages DIAMOND [76], eggNOG-mapper [77], Pfam [78], and NCBI BLAST [56]. Additional components included BUSCO [55], AGAT [79], GFFCompare [80], ViennaRNA [81], VSEARCH [82], seqtk (https://github.com/lh3/seqtk), SeqKit [83], and FeatureCounts [84]. Evidence support included Illumina RNA-seq alignments as well as protein homology to the UniProt Swiss-Prot and NCBI Plant RefSeq databases.

To optimize gene prediction in this large and highly repetitive genome, several non-default parameters were used. The -*rate* parameter, which defines the minimum RNA-seq alignment percentage required per library, was set to 80 (default = 85) to accommodate the available RNA-seq dataset, which had an alignment rate of ~ 84.8%. The -*stringency* parameter was set to 3, reducing the number of features used in Easel’s random forest–based filtering and the *-regressor* filtering threshold was lowered to 50. These adjustments were made to prevent over-filtering, as the default settings excluded a substantial number of well-supported gene models backed by both transcriptomic and homology evidence – potentially at the cost of a slightly increased false positive rate. A key concern was the potential misalignment of RNA-seq reads across repetitive regions, which can lead to spurious transcript structures and abnormally long introns. To minimize this, we explicitly limited the maximum intron length during both RNA alignment (-*hisat2_max_intronlen* = 30000) and protein mapping (-*miniprot_args* = “-G 30000”), and adjusted StringTie2 (-*stringtie2_args* = “-j 3 -f 0.2 -c 3 -M 0.95”) parameters to reduce transcript fragmentation and false isoforms.

Completeness of the predicted gene set was assessed with BUSCO v5.4.3 using the poales_odb12 lineage dataset.

### Comparative genomics and phylogenetic analysis

To place *F. glaucescens* in a phylogenetic context and validate the predicted proteome, we performed a comparative analysis using OrthoFinder v2.5.5 [85]. Protein sequences from *F. glaucescens* and selected representative Poaceae species were clustered into orthogroups. The resulting species tree was inferred by OrthoFinder from gene orthogroup relationships using default settings, including STAG [86] for species tree inference and STRIDE [87] for rooting.

## Supplementary Information

Supplementary Material 1

## Data Availability

The raw sequencing reads, genome assembly, and annotation files are available under the NCBI BioProject accession PRJNA1055418. This Whole Genome Shotgun project has been deposited at DDBJ/ENA/GenBank under the accession JBQVRU000000000. The version described in this paper is version JBQVRU010000000. The custom repeat library used for genome annotation is available on Zenodo at DOI: 10.5281/zenodo.15744278.

## Abbreviations

1Cx: Monoploid genome size
BL: Broad-leaved
FISH: Fluorescent *in Situ* Hybridization
FL: Fine-leaved
GISH: Genomic *in situ* hybridisation
GO: Gene ontology
HMW DNA: High molecular weight DNA
LINE: Long interspersed nuclear element
LTR: Long terminal repeats
rDNA: Ribosomal DNA
RFLP: Restriction fragment length polymorphism
RNA-seq: RNA sequencing
SINE: Short interspersed nuclear element
WGS: Whole-genome sequerncing
