# Supplementary material for: The draft genome assembly and annotation of allotetraploid Festuca glaucescens
Source: BMC Plant Biol. 2025 Nov 18;25:1590. doi: 10.1186/s12870-025-07558-8 (PMC12625059; doi:10.1186/s12870-025-07558-8)
Supplement: Supplementary file 1 — Supplementary Material 1 [file 12870_2025_7558_MOESM1_ESM.docx]

**Supplementary Note**: K-mer multiplicity and read depth analysis to evaluate haplotig purging

Despite the internal haplotype purging performed by HiFiAsm, the resulting assembly size (5.52 Gb) exceeded expectations based on genome size estimates (~4.6 Gb). The distribution of per-contig read depth exhibited a clear bimodal pattern (Supplementary Figure 1A), suggesting the presence of uncollapsed haplotypes. This interpretation is supported by the observation that low-depth contigs also show lower k-mer multiplicity, consistent with single-haplotype representation (Supplementary Figure 1C). While a greater number of contigs fall into the shallower depth mode, deeper contigs tend to be longer and cumulatively contribute the majority of the assembly length (Supplementary Figure 1B). Nonetheless, contigs with mean read depth <9× account for ~1.44 Gb, and collapsing these (as a proxy for haplotig removal) would reduce the total assembly size from 5.52 Gb to ~4.8 Gb, closer to the expected genome size.

We anticipated that the lack of clear separation between haplotigs and primary contigs—due to relatively shallow sequencing depth (~6.5× per haplotype)—would complicate reliable haplotig detection. Nonetheless, to assess the feasibility of automated purging, we performed an exploratory run of purge_dups (v1.2.5, default parameters, Supplementary Figure 2) on the unpurged HiFiAsm assembly. The resulting purged assembly had a reduced size of 3.72 Gb, suggesting substantial overpurging relative to the expected genome size.

Comparison of BUSCO profiles between the unpurged and purged assemblies (Supplementary Figure 3) further supports this conclusion. In an allotetraploid genome, most BUSCOs are expected to occur in two copies, one per subgenome. In the unpurged assembly, the majority of BUSCOs occur twice (5817 duplicated vs. 374 single-copy), consistent with retention of both subgenomes. After purging, two-copy BUSCOs remain the largest class, but there is a marked increase in single-copy BUSCOs (1611 vs. 374) together with a higher proportion of missing BUSCOs (2.3% vs. 0.8%) and reduced overall completeness (97.6% vs. 98.6%). While some single-copy BUSCOs are expected due to homeolog loss (fractionation) in allopolyploids, the scale of this shift—coupled with the large drop in assembled size (5.52 Gb → 3.72 Gb) relative to expectations—indicates that additional purging collapsed true homeologous copies. These results show that the purged assembly does not accurately represent the genome structure of *F. glaucescens*.

To evaluate the validity of the purging process, we compared purge_dups classifications to per-contig k-mer multiplicity (from Illumina reads) and mean read depth (from PacBio HiFi reads). While contigs flagged as duplicates showed modest enrichment in the expected region of lower depth and lower multiplicity (Supplementary Figure 4B), a large degree of overlap was observed between contigs marked as duplicates and those retained as primary (Supplementary Figure 4A). This suggests that purge_dups classifications cannot reliably distinguish true haplotigs from collapsed allelic or homeologous sequences in this dataset.

Given the lack of robust separation and the evidence of excessive sequence loss, we retained the original HiFiAsm primary assembly, which applies conservative internal purging and better preserves genome completeness and subgenomic structure.

| **Supplementary Figure 1**. Sequencing depth, contig length, and k-mer multiplicity profiles of the unpurged assembly. | |
| --- | --- |
| **a)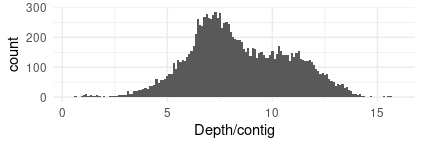** | **c)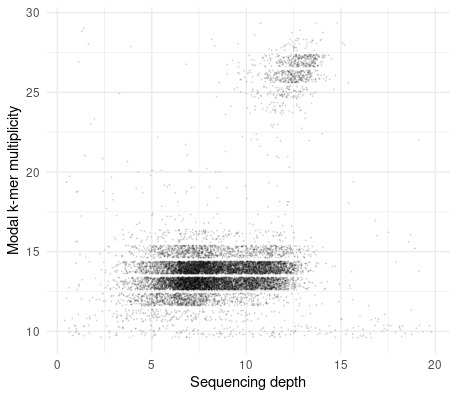** |
| **b)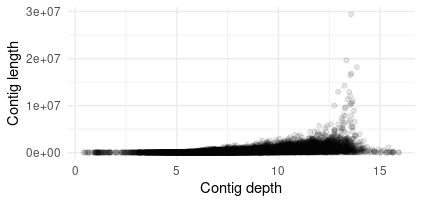** |  |
| **(a)** Histogram of mean read depth per contig, showing a clear bimodal distribution. The lower mode (~6–7×) suggests the presence of uncollapsed haplotypes, while the higher mode (~12–13×) reflects contigs where both haplotypes contribute reads.  **(b)** Scatter plot of contig length versus mean sequencing depth, showing a positive correlation between contig length and read depth. Shorter contigs tend to have lower coverage, consistent with haplotig-like sequences, while longer contigs accumulate more depth, contributing disproportionately to total assembly size.  **(c)** Scatter plot of modal k-mer multiplicity (estimated from Illumina reads) versus mean sequencing depth (based on PacBio HiFi reads) for contigs in the unpurged HiFiAsm assembly. Two main clusters are visible:   - A smaller, compact cluster around 27× multiplicity and 12–13× depth, consistent with contigs from homozygous regions, where both haplotypes contribute reads and share identical k-mers. - A larger, diffuse cluster centered around 13× multiplicity and 5–10× depth, likely representing a mixture of collapsed heterozygous or homeologous contigs (toward higher depth) and uncollapsed haplotigs (toward lower depth). | |

**Supplementary Figure** **2**. Purge_dups coverage histogram for the *F. glaucescens* assembly. The blue curve shows the read depth distribution across the assembly. Vertical lines indicate automatically inferred coverage cutoffs (red = low, green = repeat, cyan = high). At the available coverage (~6.5× per haplotype), the peaks are not clearly separated, complicating reliable purging.

| 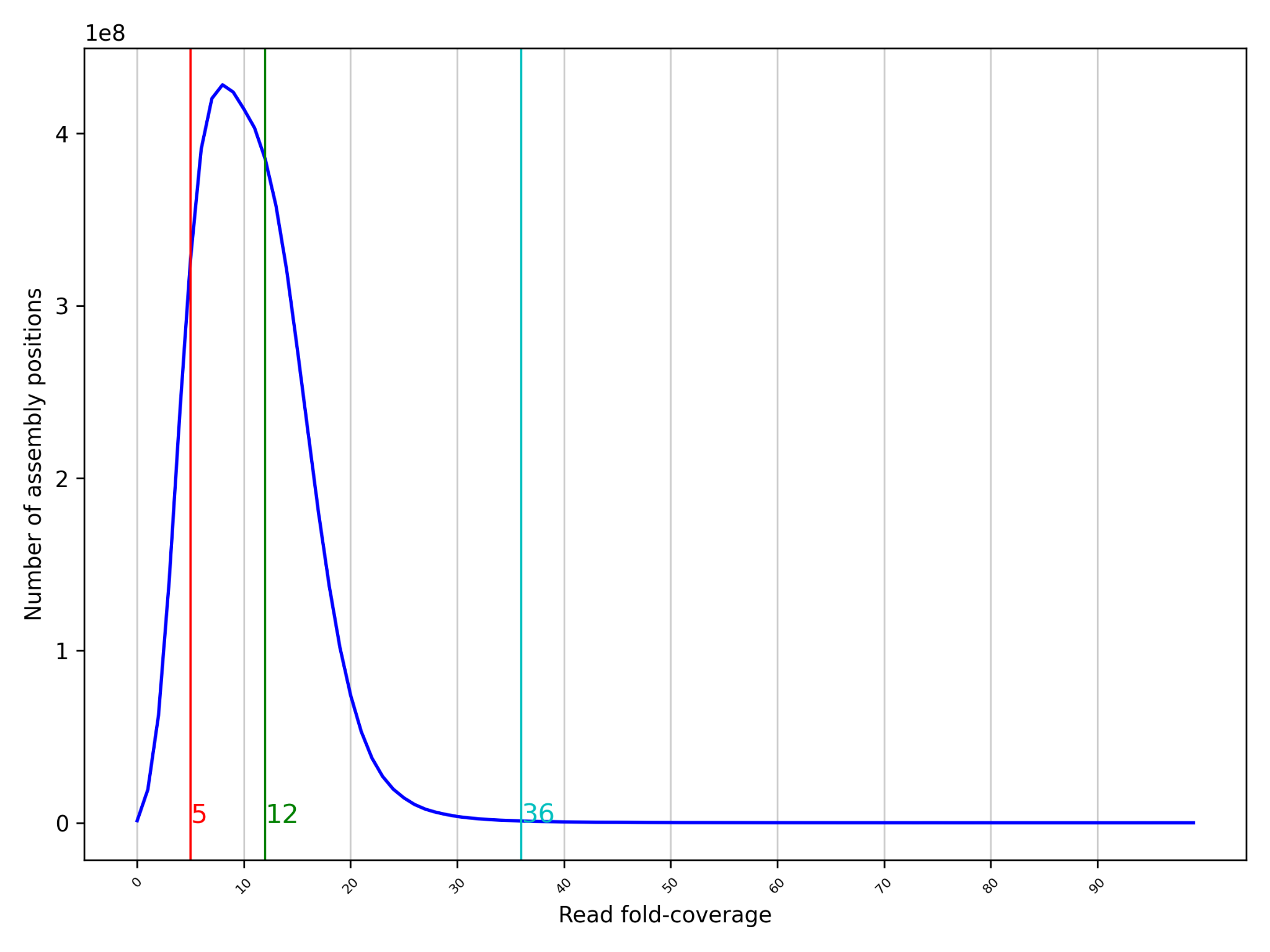  **Supplementary Figure 3**. BUSCO comparison between the unpurged and purge_dups-purged assemblies.   \| **a)**  **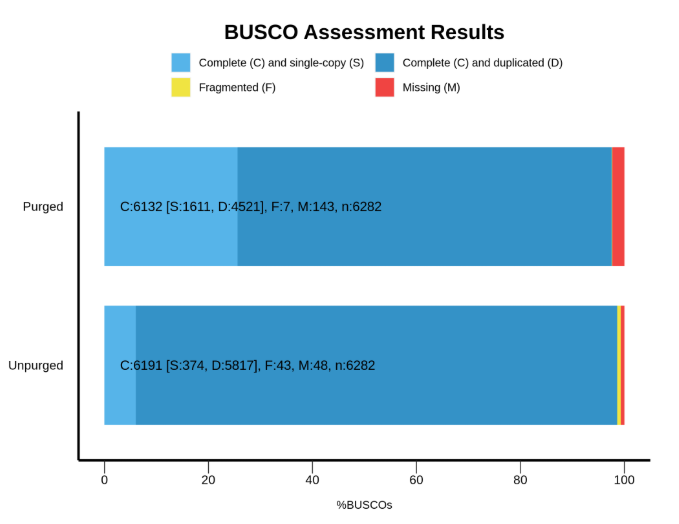** \| **b)**  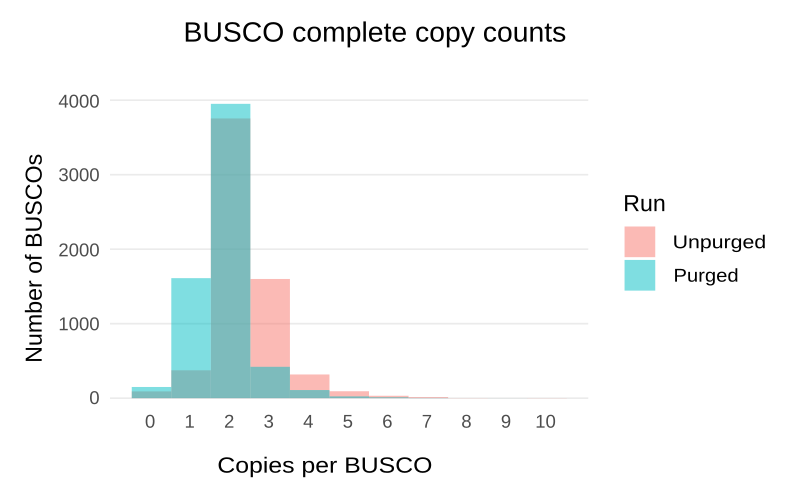 \| \| --- \| --- \|   **(a)** BUSCO completeness profiles (poales_odb12, n = 6282). The purged assembly shows reduced overall completeness (97.6% vs. 98.6%), more missing BUSCOs (2.3% vs. 0.8%), and a clear shift from duplicated to single-copy BUSCOs (1611 vs. 374 single-copy).  **(b)** Copy-number distribution of complete BUSCOs. Both assemblies are dominated by BUSCOs in two copies, as expected for an allotetraploid genome. Compared to the unpurged assembly, the purged assembly has fewer ≥3-copy BUSCOs (haplotigs removed) but more single-copy BUSCOs, suggesting that haplotig collapse came at the cost of losing true homeologous copies.  **Supplementary Figure 4**. K-mer multiplicity and read depth profiles of contigs classified by purge_dups. | |
| --- | --- | --- | --- |
| **a)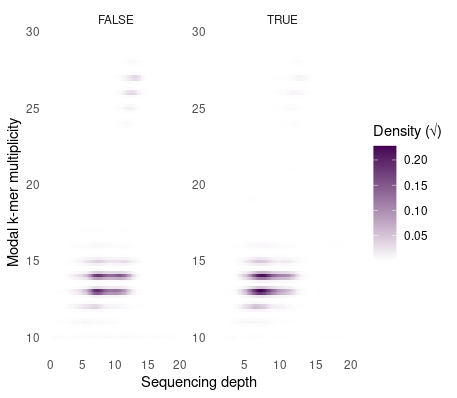** | **b)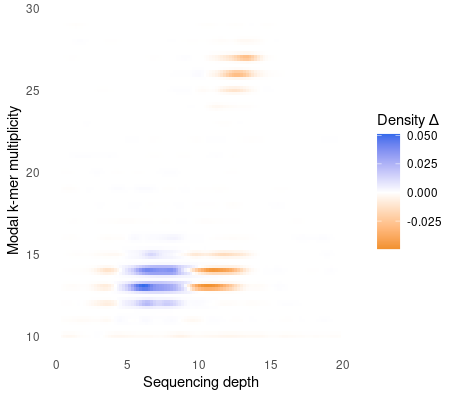** |
| **(a)** 2D density plots of modal k-mer multiplicity (from Illumina reads) versus mean sequencing depth (from PacBio HiFi reads) for contigs classified as duplicated (TRUE) or non-duplicated (FALSE) by purge_dups. Both groups exhibit overlapping distributions centered around ~13× multiplicity and ~5–10× depth, with no clear separation between primary and duplicate contigs.  **(b)** A differential 2D density plot (duplicate minus non-duplicate) highlights regions with relative enrichment of contigs labeled as duplicates (blue) or non-duplicates (orange). While some enrichment of duplicates is seen in the low-depth, low-multiplicity region—consistent with uncollapsed haplotigs—these differences are subtle. The majority of duplicate-labeled contigs occupy the same signal space as primary contigs. | |
